# The Interaction of Endothelin-1 and TGF-β1 Mediates Vascular Cell Remodeling

**DOI:** 10.1371/journal.pone.0073399

**Published:** 2013-08-28

**Authors:** Christopher Lambers, Michael Roth, Jun Zhong, Christoph Campregher, Petra Binder, Bernhard Burian, Ventzislav Petkov, Lutz-Henning Block

**Affiliations:** 1 Division of Respiratory Medicine, Department of Internal Medicine II, Medical University of Vienna, Vienna, Austria; 2 Pulmonary Cell Research/Pneumologie, Department Biomedicine/Internal Medicine, University Basel/University Hospital, Basel, Basel, Switzerland; 3 Christian Doppler Laboratory for Molecular Cancer Chemoprevention, Division of Gastroenterology and Hepatology, Department of Medicine 3, Medical University of Vienna, Vienna, Austria; Albert Einstein College of Medicine, United States of America

## Abstract

**Background:**

Pulmonary arterial hypertension is characterized by increased thickness of pulmonary vessel walls due to both increased proliferation of pulmonary arterial smooth muscle cell (PASMC) and deposition of extracellular matrix. In patients suffering from pulmonary arterial hypertension, endothelin-1 (ET-1) synthesis is up-regulated and may increase PASMC activity and vessel wall remodeling through transforming growth factor beta-1 (TGF-β1) and connective tissue growth factor.

**Objective:**

To assess the signaling pathway leading to ET-1 induced proliferation and extracellular matrix deposition by human PASMC.

**Methods:**

PASMC were serum starved for 24 hours before stimulation with either ET-1 and/or TGF-β1. ET-1 was inhibited by Bosentan, ERK1/2 mitogen activated protein kinase (MAPK) was inhibited by U0126 and p38 MAPK was inhibited by SB203580.

**Results:**

ET-1 increased PASMC proliferation when combined with serum. This effect involved the mitogen activated protein kinases (MAPK) ERK1/2 MAPK and was abrogated by Bosentan which caused a G1- arrest through activation of p27^(Kip)^. Regarding the contribution of extracellular matrix deposition in vessel wall remodeling, TGF-β1 increased the deposition of collagen type-I and fibronectin, which was further increased when ET-1 was added mainly through ERK1/2 MAPK. In contrast, collagen type-IV was not affected by ET-1. Bosentan dose-dependently reduced the stimulatory effect of ET-1 on collagen type-I and fibronectin, but had no effect on TGF-β1.

**Conclusion and Clinical Relevance:**

ET-1 alone does not induce PASMC proliferation and extracellular matrix deposition. However, ET-1 significantly up-regulates serum induced proliferation and TGF-β1 induced extracellular matrix deposition, specifically of collagen type-I and fibronectin. The synergistic effects of ET-1 on serum and TGF-β1 involve ERK1/2 MAPK and may thus present a novel mode of action in the pathogenesis of pulmonary arterial hypertension.

## Introduction

Pulmonary arterial hypertension (PAH) is characterized by the progressive increase of pulmonary vascular resistance and pulmonary arterial pressure, leading to right heart failure and death [1,2]. PAH is either a disease on its own or occurs as co-morbidity in patients suffering from hypoxia, scleroderma, or chronic obstructive pulmonary disease (COPD) [2,3]. A major pathology of PAH is the structural change of the pulmonary arterial vessel wall, which is expressed as hypertrophic smooth muscle and increased deposition of extracellular matrix (ECM) in the tunica media [4,5]. Both events are independent of each other and result in the narrowing of the lumen of pulmonary arteries. The thickened vessel wall is caused by increased deposition of ECM and by smooth muscle hyperplasia/hypertrophy; together these pathologies reduce the flexibility of the vessel wall and increase the constrictive force of the muscle bundles, thereby increasing the pulmonary arterial blood pressure [5].

Endothelin-1 (ET-1) plasma levels are prominently increased in PAH patients and correlate with pulmonary vascular resistance, pulmonary arterial pressure, cardiac index, and cardiac output [6,7]. ET-1 binds and activates the G-protein coupled ET_A_ and ET_B_ receptors and thereby increase intracellular calcium levels, which in turn activate both phospholipase C and protein kinase C [8–11]. Both ET-receptors mediate vasoconstriction and stimulate pulmonary arterial smooth muscle cell (PASMC) proliferation [10–12]. The mechanism underlying the ET-1 dependent thickening of the pulmonary arterial wall involves the increase of intracellular calcium, cAMP generation and subsequent up-regulation of cyclo-oxygenase 2 in an autocrine loop [13]. There is evidence that the distribution of the ET-receptors is cell type and species-specific; therefore, data obtained in PAH animal models cannot be directly compared to the pathology of human PAH [14].

In human lung fibroblasts and vascular smooth muscle cells, ET-1 activates at least one mitogen-activated protein kinases (MAPK), ERK1/2, which in turn activates cyclins and cyclin-dependent kinases resulting in cell proliferation and subsequently PASMC hyperplasia in PAH [15,16]. MAPK also mediate the increase of ECM deposition in the vessel wall of PAH patients [17,18]. Moreover, PAH is associated with an altered interaction of MAPK with the transforming growth factor β1 (TGF-β1) activated signaling cascades, which are relevant to ECM restructuring [17,19]. Increased expression of TGF-β1 and connective tissue growth factor (CTGF) have been observed in PAH vessel and contribute to PASMC growth and collagen deposition [17,20]. Taking into account that the collagen content of pulmonary arteries has been suggested as an indicator of PAH staging and progression, the role of TGF-β1 and CTGF, together with other disease relevant stimuli, such as ET-1 on this pathology, has to be further investigated [21]. However, it is not known which specific components of the ECM participate in PAH vessel wall remodeling.

In this study, we determined the effect of ET-1 alone as well as in combination with a ubiquitous mitogen and TGF-β1 on human primary PASMC. We determined the contribution of ET-1 and its receptors on TGF-β1 induced MAPK signaling, proliferation and ECM metabolism in human primary PASMC.

## Materials and Methods

### Cell culture and proliferation

Experiments were performed using a commercially available cell line of primary human PASMC (CC-2581, Cambrex-Bioscience, Walkersville, USA), which was grown as recommended by the manufacturer. Experiments were performed when the monolayer of PASMC reached 70%-80% confluence. Prior to experiments, PASMC were serum-deprived in medium containing 0.5% fetal calf serum (FCS) for 24 hours. ET-1 (Sigma, Vienna, Austria) was used in a concentration range of 0.01-1 µM. Bosentan (Tracleer™, Actelion Pharmaceuticals, Allschwil, Switzerland) was used at 10 and 100 µM and was added to cell cultures 15 minutes before stimulation. FCS (5%) was used as a positive control.

Cell proliferation was determined by direct cell count after 3 days of incubation [22].

### Immunofluorescence microscopy

PASMC were grown on chamber slides and treated with ET-1 and/or Bosentan for various time periods before fixation in methanol (15 minutes). Slides were washed 3x with phosphate buffered saline (PBS), then incubated with blocking buffer (10% BSA in PBS) for 20 minutes at room temperature (RT), followed by incubation (2 hours, RT) with a monoclonal antibody specific to phosphorylated amino acids Thr 202 and Tyr 204 of ERK1/2 (#9106, diluted 1:400, Cell Signaling Technology, USA). Slides were washed 5x with PBS and incubated (45 minutes, RT) with a FITC-conjugated species specific second antibody (Sigma). Propidium iodide (15 minutes) was used to stain nuclei. Slides were washed 3x with blocking buffer and evaluated by fluorescence microscope (Olympus, BX41, Vienna, Austria).

### Proteins: Whole cell, nuclear and cytosolic

PASMC were harvested in ice cold PBS and centrifuged (1000xg, 6 minutes). Whole cell lysates where prepared by resuspension of the cell pellet in lysis buffer [50 mM/L TrisCl pH 7.4, 150 mM NaCl, 1mM EDTA pH 8, 1% NP-40, complete protease inhibitor cocktail^TM^ (Roche Diagnostics, Vienna, Austria)] and frozen twice in liquid nitrogen and incubated on ice (20 minutes). The supernatant was collected after centrifugation (13,000×g, 15 minutes, 4°C).

Cytoplasmic proteins were extracted from cells in low-salt buffer (20 mM HEPES pH 7.9, 10 mM KCl, 1 mM EDTA, 1 mM EGTA, 0.2% NP-40, 10% glycerol complete protease inhibitor^TM^ (Roche Diagnostics, Vienna, Austria) and incubated on ice for 10 minutes. After centrifugation (13,000xg, 1 minute, 4°C), the supernatant was collected as cytosolic protein fraction. The remaining pellet was dissolved in high-salt buffer (420 mM NaCl, 20 mM HEPES pH 7.9, 10 mM KCl, 1 mM EDTA, 1 mM EGTA, 20% glycerol, Complete Protease Inhibitor™) and incubated on ice (30 minutes), followed by centrifugation (13,000xg, 10 minutes, 4°C). The supernatant was collected as nuclear protein fraction. Protein concentrations were determined by Bradford assay (Bio-Rad, Vienna, Austria) at 595 nm. In experiments for protein phosphorylation studies, we added PhosSTOP buffer (Roche Diagnostics) to protein samples.

### Immunoblot analysis

Proteins (25 μg) were size fractionated by gel electrophoresis (12% SDS-polyacrylamide gel), then transferred onto a PVDF membrane (Millipore Corp., Billerica, USA) by standard protocols. The transfer was confirmed by Ponceau red staining [22]. Membranes were incubated (2 hours, RT) in blocking buffer (PBS, 0.1% Tween-20, 5% skimmed milk) before a protein-specific monoclonal antibody was added for overnight incubation (4°C). The membranes were washed 3x (80 nM Na_2_HPO_4_, 20 mM NaH_2_PO_4_, 100 mM NaCl, 0.1% Tween-20, pH 7.5) and then incubated (90 minutes, RT) with a secondary horse radish peroxidase-labeled species specific antibody (Amersham, Buckinghamshire, UK). After being washed 3x with PBS, the protein bands were visualized by enhanced chemiluminescence substrate (Pierce, Illinois, USA) and documented on X-ray film for image analysis (ImageJ version 1.37). Antibodies used: ETA receptor (N-15, Santa Cruz), ETB receptor (A-20, Santa Cruz), actin (Sigma, A-2066), p27^(Kip1)^ (AB3003, Chemicon).

### ECM, collagen and fibronectin deposition

Deposition of total ECM was determined in confluent cell layers. For total ECM, cells were starved for 24 hours before being stimulated with either ET-1 or TGF-β1 for 48 hours. [^3^H]-proline (1 μM Ci) was added for the last 24 hours before cells were washed with PBS followed by fixation in ice cold methanol: acetic acid (3:1) for 5 minutes and subsequent measuring of ECM incorporated tritium as described earlier [23].

The deposition of collagen type-I, -III and -IV were determined by a modified enzyme linked immunosorbent assay as described earlier [24]. In brief, PASMC were seeded into a 96-well plate and grown to confluence before being stimulated with ET-1 (1 µM) for 48 and 72 hours. Cells were removed by 1 hour incubation in hypotonic ammonium hydroxide. Plates were incubated with 2% bovine serum albumin (30 minutes), followed by overnight incubation (4^o^C) with one of the primary anti-bodies (collagen type-I: sc-59772, type-III: sc-8781, type-IV: sc-52317, fibronectin: sc-271098; all from Santa Cruz Biotech (Santa Cruz, USA)). Plates were washed 5x with PBS and incubated with a secondary horse radish peroxidase labeled species specific antibody (1 hour, RT). Plates were washed 5x with PBS and 100 μl of TMB solution were applied to each well for 30 minutes. The signal was determined by ELISA reader.

For experiments with neutralizing antibodies targeting pan-TGF-β and CTGF (R&D systems, Abingdon, UK), cells were pre-incubated for 30 minutes before stimulation.

### Real-time PCR

Total mRNA was isolated by TRIZOL [25] and transcribed into cDNA by reverse transcriptase (Advantage RT-for-PCR Kit, Clontech, Palo Alto, USA). Real-time PCR was performed by LightCycler480 (Roche Diagnostics) using the following commercially available primers: p27^(Kip1)^ mRNA (Hs00153277_m1), TGF-β1(Hs00171257_m1) and collagen type 1 alpha1 chain (Hs01076777_m1); all were purchased as TaqMan pre-developed gene expression assay (Applied Biosystems, ABI, CA, USA). The internal control for PCR analysis was 18S mRNA (Applied Biosystems). 50 PCR cycles (95°C for 10 seconds; 65°C for 30 seconds; 72°C for 5 seconds) were performed. Relative mRNA expression was determined using the ΔCT method [26].

### Cell cycle phase characterization

PASMC (1 x 10^5^ cells/ml) were grown in 6-well plates and stimulated by ET-1 (0.1-1 µM) in the presence or absence of Bosentan (100 µM) for various time intervals. FCS (5%) was used as positive control. Cells were harvested after 6 and 24 hours by accutase treatment, washed with PBS, and fixed in 100% ethanol (30 minutes, on ice). Cells were centrifuged (1500 rpm, 5 minutes) and re-suspended in PBS-containing 20 µg/ml propidium iodide (Sigma) and 60 µg/ml RNase A (Sigma) for 15 minutes (RT). The DNA content was determined by flow cytometry, and the cell cycle distribution was analyzed by CellQuest Software (Becton Dickinson, Mountain View, CA).

### Statistics

Data are presented as mean ± S.E.M. and the Null-hypothesis was: no difference between treated and untreated cells. Protein expression and mRNA transcription were compared by Student’s t-test (two-tailed, unpaired). The effect of Bosentan on ET-1 induced cell cycle progression was compared by multivariate analysis ANOVA. Results were considered significant when the p-value was < 0.05.

## Results

### Effects of endothelin-1 on cell proliferation

**Figure 1:**
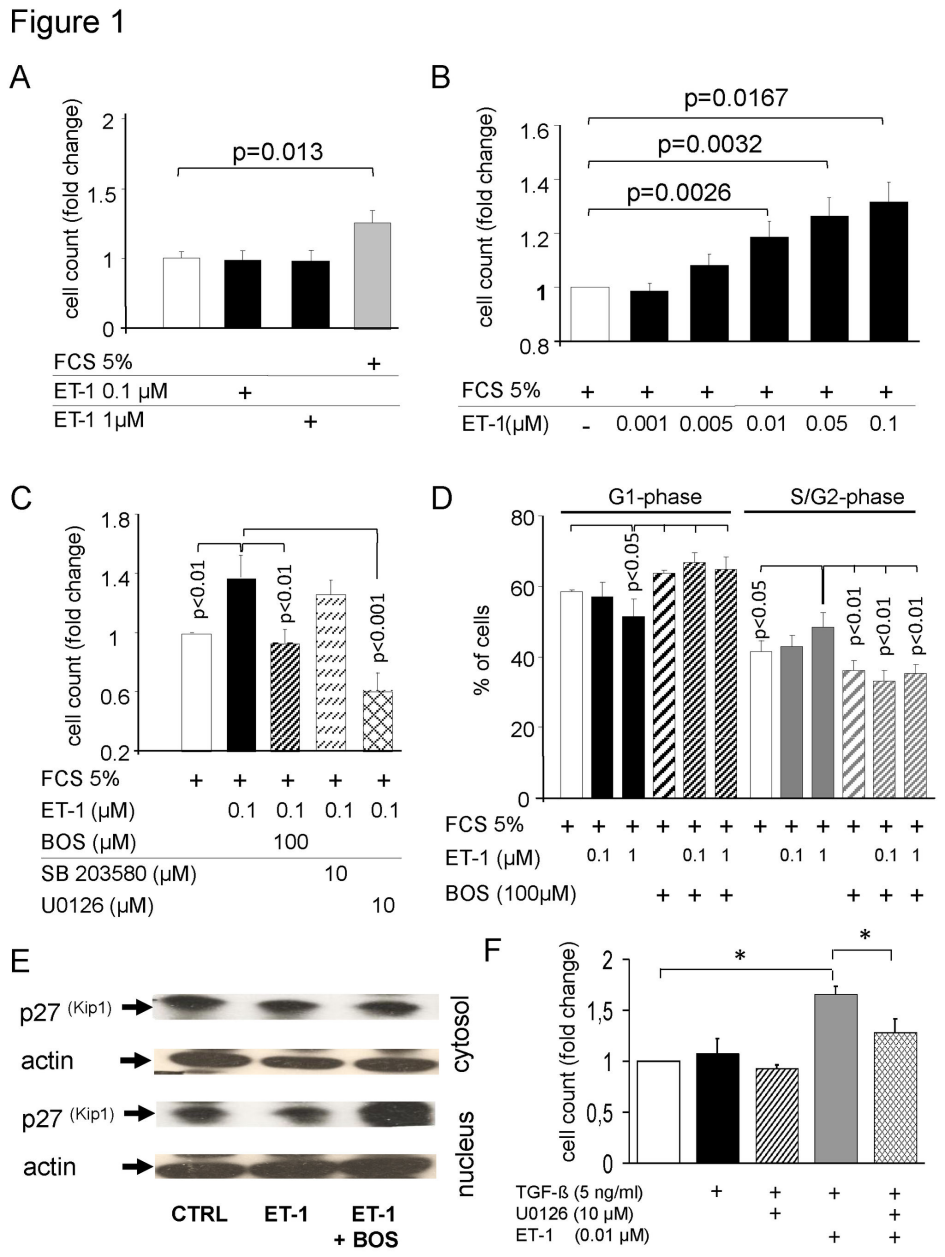
The mitogenic-supportive effect of Et-1 on PASMC. (A) ET-1 alone has no mitogenic effect, while 5% foetal calf serum (FCS) induced proliferation of PASMC within 3 days (n = 6). (B) When combined with 5% FCS, ET-1 dose-dependently increased cell numbers within 3 days (n = 6). Similar results were obtained after 5 days. (C) ET-receptor blockade by Bosentan and chemical Erk1/2 MAPK inhibition abolished the supportive effect of ET-1 on serum-induced PASMC proliferation (n = 3). (D) ET-1 induced accumulation of S/G2-phase cells is prevented by Bosentan (n = 3). (A–D) Bars represent mean ± SEM. (E) Representative immune-blot of the effect of ET-1 and Bosentan on cell compartment distribution of p27^(Kip)^, similar results were obtained in 3 additional experiments. (F) TGF-ß alone did not induce proliferation, whereas the addition of ET-1 significantly increased PASMC proliferation, which was abolished by the inhibition of Erk1/2 MAPK.

ET-1 (0.1-1 μM) did not induce proliferation of growth arrested, serum starved PASMC; while 5% FCS stimulation increased the cell number by ~1.8 fold over 3 days (Figure 1A). When combined, ET-1 dose-dependently further increased the FCS (5%) induced proliferation of PASMC up to 1.43 fold (Figure 1B). Bosentan (100 μM) significantly reduced the ET-1 induced proliferation of PASMCs (Figure 1C). The p38 MAPK inhibitor SB203580 (10 µM) had no significant effect on ET-1 induced PASMC proliferation, while the ERK1/2 inhibitor U0126 significantly reduced both ET-1 and 5% FCS-induced proliferation (Figure 1C). To further characterize the effect of ET-1 on proliferation control, we determined the cell cycle distribution and found that ET-1 reduced the number of cells in the G1-phase in a dose-dependent manner, while it increased the number of cells in G2-phase from 39.2% to 48.6%. When pre-incubated with Bosentan, the percentage of cells in the G2-phase significantly decreased to 33.5% (Figure 1D). The inhibitory effect of Bosentan on ET-1 induced proliferation was paralleled by increased nuclear accumulation of the cell cycle inhibitor p27^(Kip1)^ (Figure 1E) while p21^(Waf1/Cip1)^ was not affected (data not shown). TGF-ß (0, 0.5, 1 and 5 ng/ml) had no effect on proliferation (data only shown for 5 ng/ml, Figure 1F). In contrast, the addition of ET-1 (10 nM) significantly induced proliferation, while the ERK1/2 inhibitor U0126 significantly reduced the combined effect of ET-1 and TGF-ß-induced proliferation (Figure 1F).

### The role of ERK1/2 MAP-Kinase in ET-1 induced proliferation

**Figure 2:**
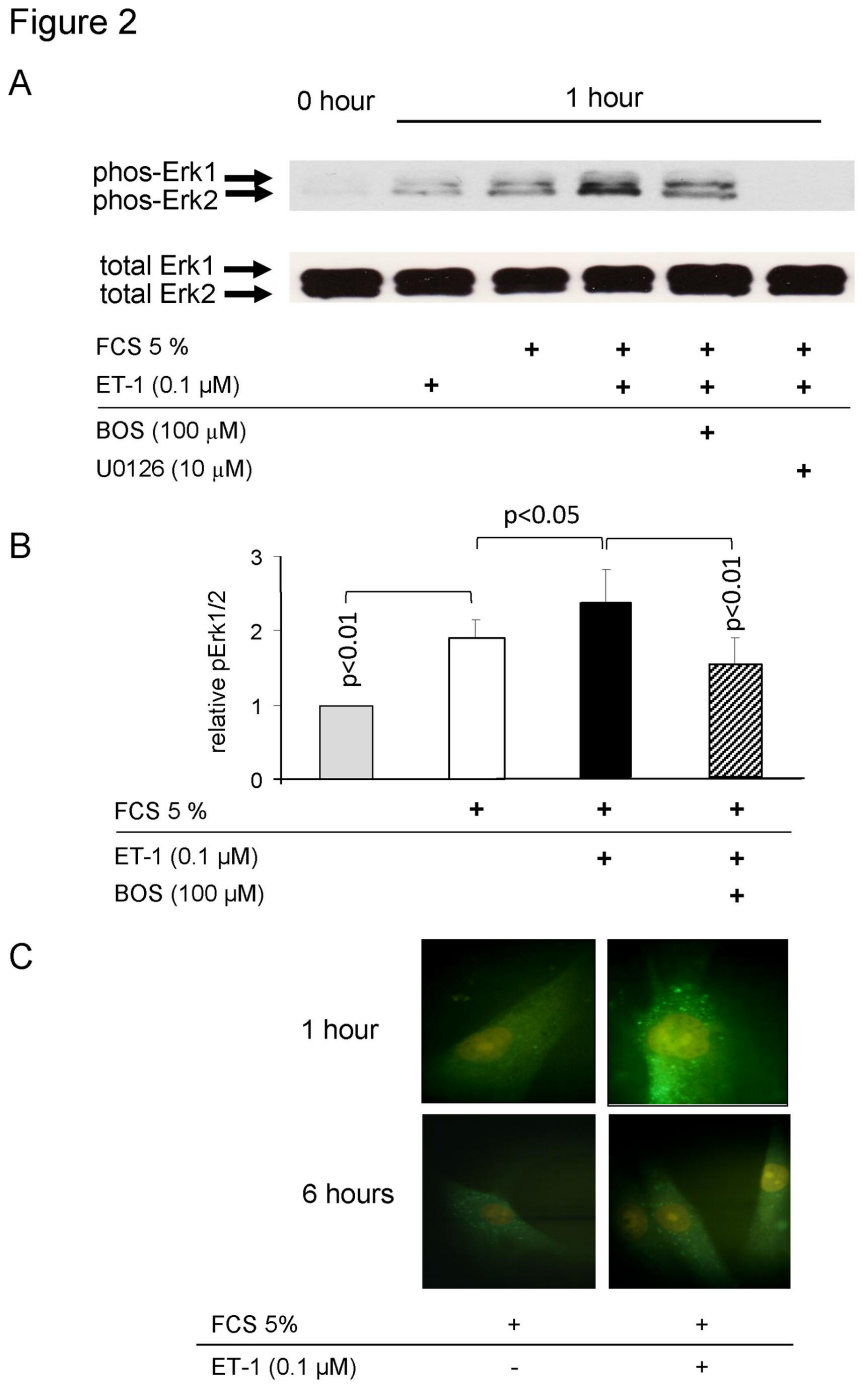
ET-1 activates and Bosentan prevents Erk1/2 MAPK activation. (A) Representative immune-blot of Erk1/2 MAPK phosphorylation (phos-Erk1, phos-Erk2). ET-1 and 5% FCS activate phosphorylation of Erk1/2 MAPK, and Bosentan prevents the additive effect of ET-1 dependent Erk1 and Erk2 phosphorylation, while the Erk172 inhibitor U0126 prevents all Erk1/2 phosphorylation. Similar experiments were performed in three additional experiments, and the result of all four experiments are presented as bar chart (mean ± SEM) in panel B. (C) The ET-1 supportive effect on serum induced nuclear accumulation of Erk1/2MAPK (representative microscopy of 5 different experiments).

In human PASMC, 5% FCS significantly induced the phosphorylation of ERK1/2 MAPK by 1.9 fold at 60 minutes; while the content of total ERK1/2 MAPK was not changed (Figure 2A). Addition of ET-1 to 5% FCS further increased the phosphorylation of ERK1/2 MAPK, and this effect of ET-1 was prevented by 30 minutes pre-incubation with Bosentan (Figure 2A). In the presence of U0126, the phosphorylation of ERK1/2 MAPK was completely suppressed (Figure 2A). The results from triplicate experiments for all conditions were quantified by image analysis and are presented as bar charts in Figure 2B. The nuclear accumulation of ERK1/2 in the presence of ET-1 has also been demonstrated by immune-fluorescence microscopy (Figure 2C).

### ET-1 alters extracellular matrix synthesis through TGF-β1 but not through CTGF

**Figure 3:**
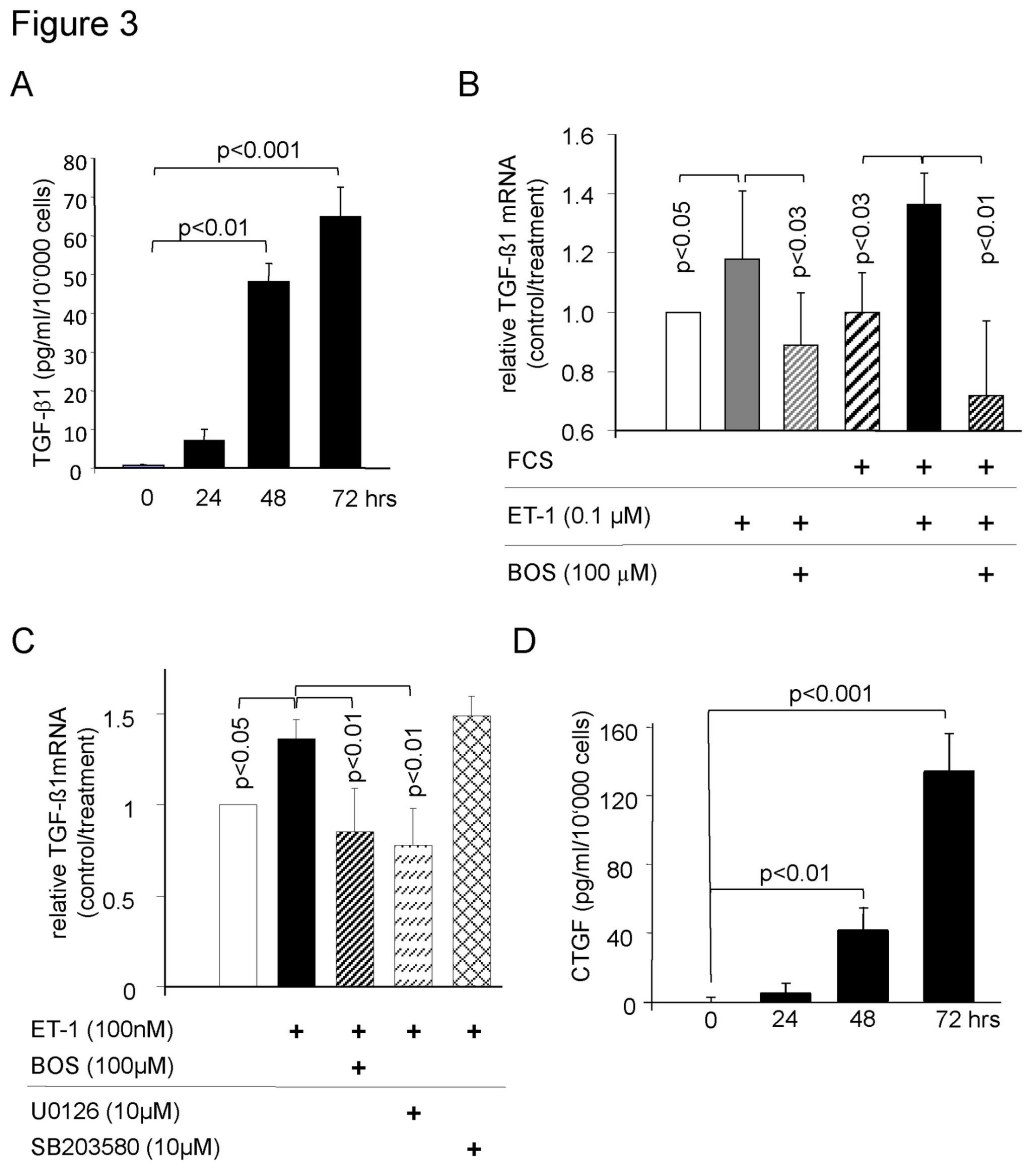
ET-1 stimulates *de novo* synthesis and secretion of TGF-β1 and CTGF. (A) TGF-β1 secretion by PASMC stimulated with ET-1 (1 μM) over 72 hours (n = 3; mean ± SEM). (B) The effect of ET-1 with and without 5% FCS and the inhibitory effect of Bosentan on TGF-β1 mRNA synthesis over 24 hours (n = 4; mean ± SEM). (C) Erk1/2 but not p38 MAPK, mediates ET-1 induced mRNA synthesis of TGF-β1 by PASMC over 24 hours (n = 4; mean ± SEM). (D) ET-1 induced CTGF secretion over 72 hours (n = 3; mean ± SEM).

Tissue remodeling in PAH also involves increased ECM deposition. Therefore, we assessed the effect of ET-1 alone or in combination with the fibrosis relevant stimulus TGF-β1 in PASMC. As shown in Figure 3A, ET-1 stimulated the secretion of TGF-β1 by PASMC over 3 days continuously; this effect of ET-1 was prevented when the cells were pre-incubated for 30 minutes with Bosentan. This was confirmed on the mRNA level (Figure 3B). When combined with 5% serum, TGF-β1 mRNA levels were further increased while Bosentan significantly reduced ET-1 and serum induced TGF-β1 mRNA levels as shown in Figure 3B. Regarding the role of MAPK in ET-1 signaling, we provide data in Figure 3C that UO126 but not SB203580 inhibited ET-1 induced TGF-β1 mRNA *de novo* synthesis. We further observed that ET-1 stimulation increased the secretion of CTGF by PASMC and pre-incubation with Bosentan prevented this effect (Figure 3D).

**Figure 4:**
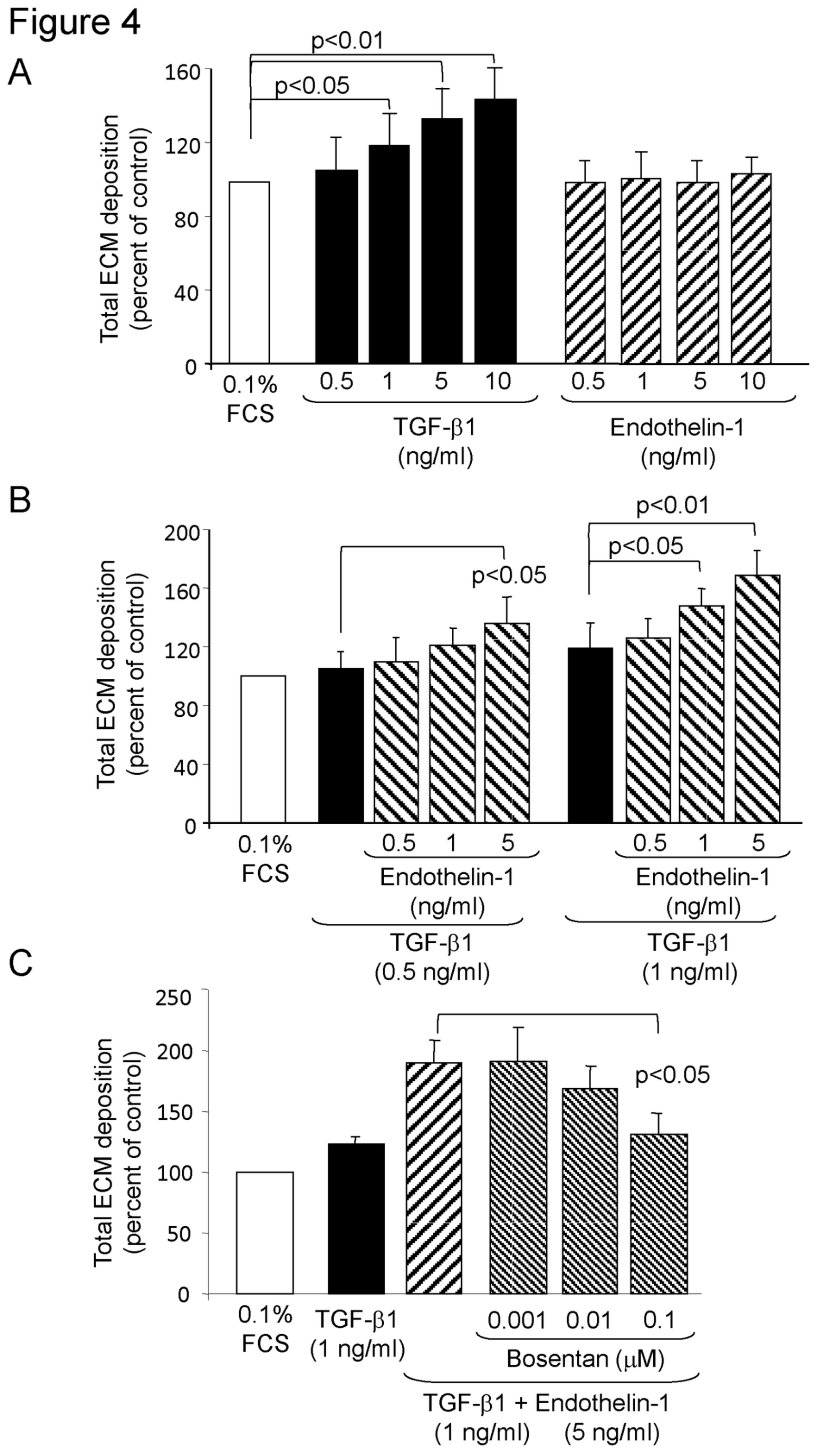
ET-1 supports the ECM stimulatory effect of TGF-β1. (A) Dose-dependent effect of 48 hours stimulation with TGF-β1 or ET-1 on total ECM deposition determined by [^3^H]-proline incorporation over the final 24 hours (n = 4; mean ± SEM). (B) Dose-dependent supportive effect of ET-1 on TGF-β1-induced total ECM deposition over 48 hours demonstrated by two fixed concentrations of TGF-β1 (n = 4; mean ± SEM). (C) Bosentan inhibits the supportive effect of ET-1 on TGF-β1 induced total ECM deposition, but has no effect on TGF-β1-induced ECM (n = 4; mean ± SEM).

Next we determined the effect of ET-1 on ECM deposition. TGF-β1 but not ET-1 dose dependently increased the deposition of total collagens by PASMC within 2 days as shown in Figure 4A. When combined, ET-1 dose dependently increased the collagen stimulating effect of TGF-β1 (Figure 4B). When the cells were pre-incubated with Bosentan, the drug dose dependently prevented the synergistic effect of ET-1 on TGF-β1 dependent collagen deposition, but it had no effect of TGF-β1 itself (Figure 4C).

**Figure 5:**
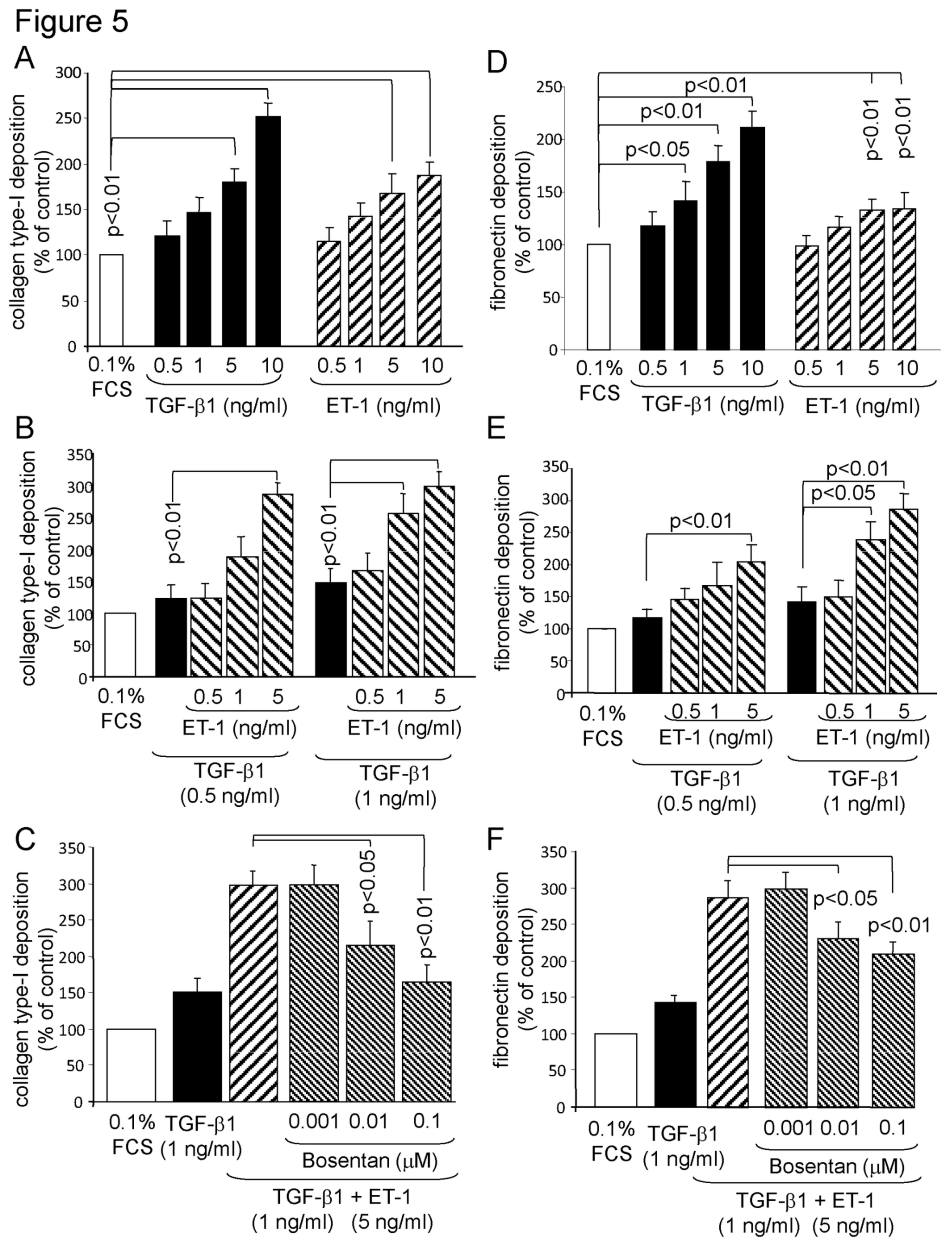
The supportive effect of ET-1 on TGF-β1 induced collagen type-I and fibronectin deposition is prevented by Bosentan. (A) Dose-dependency of TGF-β1 and ET-1 on collagen type-I deposition at 48 hours (n = 4; mean ± SEM). (B) The supportive effect of ET-1 on TGF-β1-induced collagen type-I deposition (n =4; mean ± SEM). (C) Bosentan prevents ET-1 support on TGF-β1 induced collagen type-I deposition (n = 4; mean ± SEM). (D) Dose-dependency of TGF-β1 and ET-1 on fibronectin deposition at 24 hours (n = 4; mean ± SEM). (E) ET-1 supports TGF-β1-induced fibronectin deposition (n =4; mean ± SEM). (F) The preventive effect of Bosentan on ET-1- support on TGF-β1 induced fibronectin deposition (n = 4; mean ± SEM).

We further investigated the effect of ET-1 on ECM composition and we observed a significant dose dependent increase of collagen type-I by both TGF-β1 and ET-1 (Figure 5A). When combined, the two stimuli had an additive effect on collagen type-I deposition, which was dose dependent for ET-1 (Figure 5B). This additive effect of ET-1 on collagen type-I deposition was dose dependently reduced when the cells were pre-incubated with Bosentan (Figure 5C).

**Figure 6:**
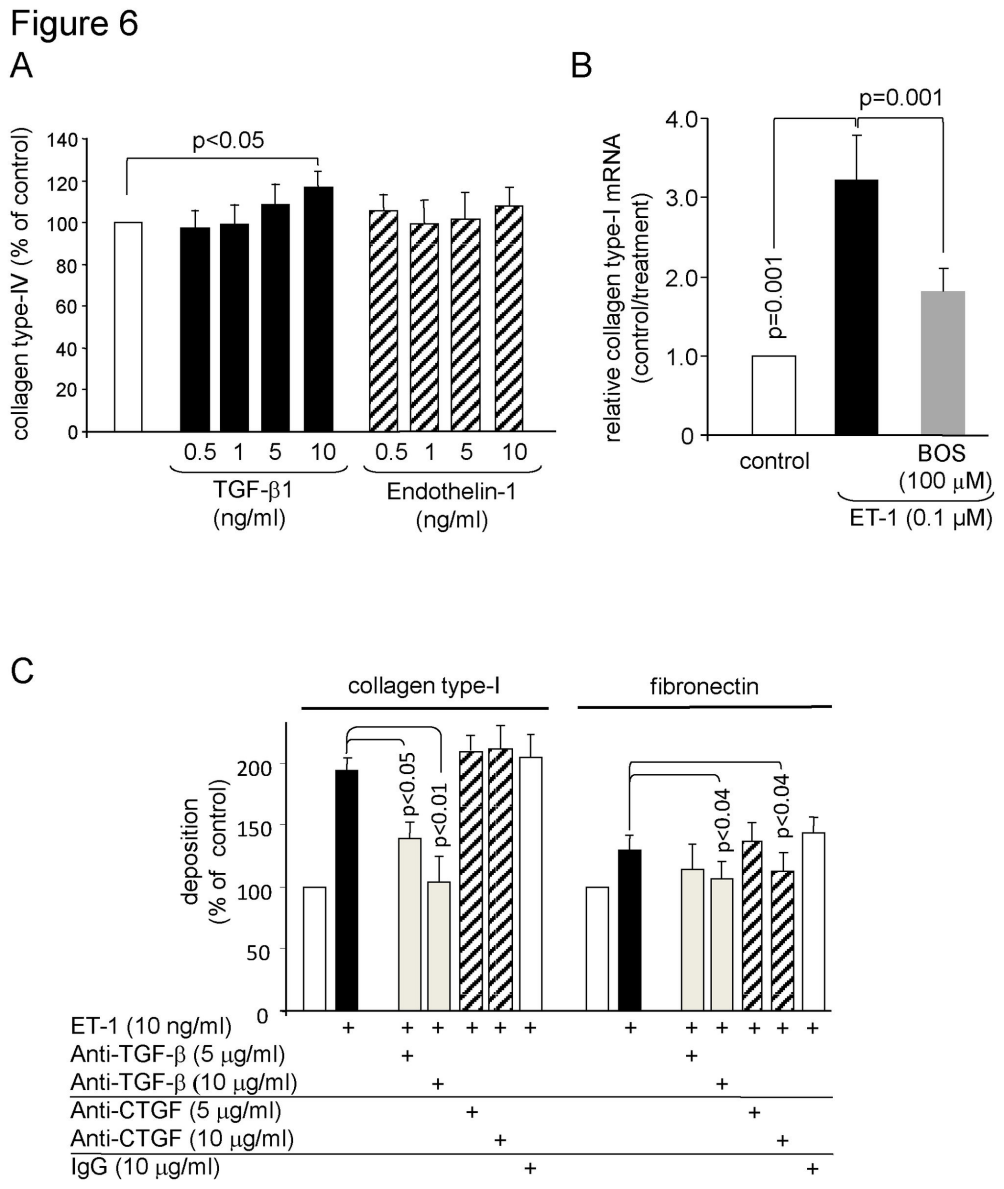
Other collagens and the stimulatory effect of ET-1 on collagen type-I *de novo* synthesis by human PASMC. (A) TGF-β1 but not ET-1 increases collagen type-IV deposition by PASMC (n =4; mean ± SEM). (B) Bosentan inhibits the ET-1 induced *de novo* synthesis of collagen type-I (n =4; mean ± SEM). (B) The involvement of TGF-β1 and CTGF in ET-1 induced collagen type-I and fibronectin deposition was investigated in the presence of neutralizing anti TGF-β and anti CTGF antibodies over 48 hours (n = 3; mean ± SEM).

Fibronectin also contributes to inflammation and was up-regulated significantly in a dose dependent manner by TGF-β1 and ET-1, with the ET-1 being less effective than TGF-β1 (Figure 5D). When the two stimuli were combined, ET-1 synergistically increased the stimulatory effect of TGF-β1 on fibronectin deposition (Figure 5E). Pre-incubation of the cells with Bosentan reduced the synergistic effect of ET-1 on fibronectin deposition; however, the inhibitory efficacy was not as strong as observed for collagen type-I (Figure 5F). We also assessed the effect of ET-1 alone and in combination of TGF-β1 on the deposition of collagen type-IV, which was not significantly affected by ET-1 (Figure 6A).

The reducing effect of Bosentan on collagen type-I deposition occurs on the transcription level as shown in Figure 6B. Pre-incubation of the cells with either TGF-β1 or CTGF neutralizing antibodies over 24 hours significantly reduced the ET-1 stimulatory effect on collagen type-I and fibronectin. In figure 6C, we provide evidence that the ET-1 induced deposition of collagen type-I was mediated through TGF-β1 alone, while its effect on fibronectin involves both TGF-β1 and CTGF.

## Discussion and Conclusions

**Figure 7:**
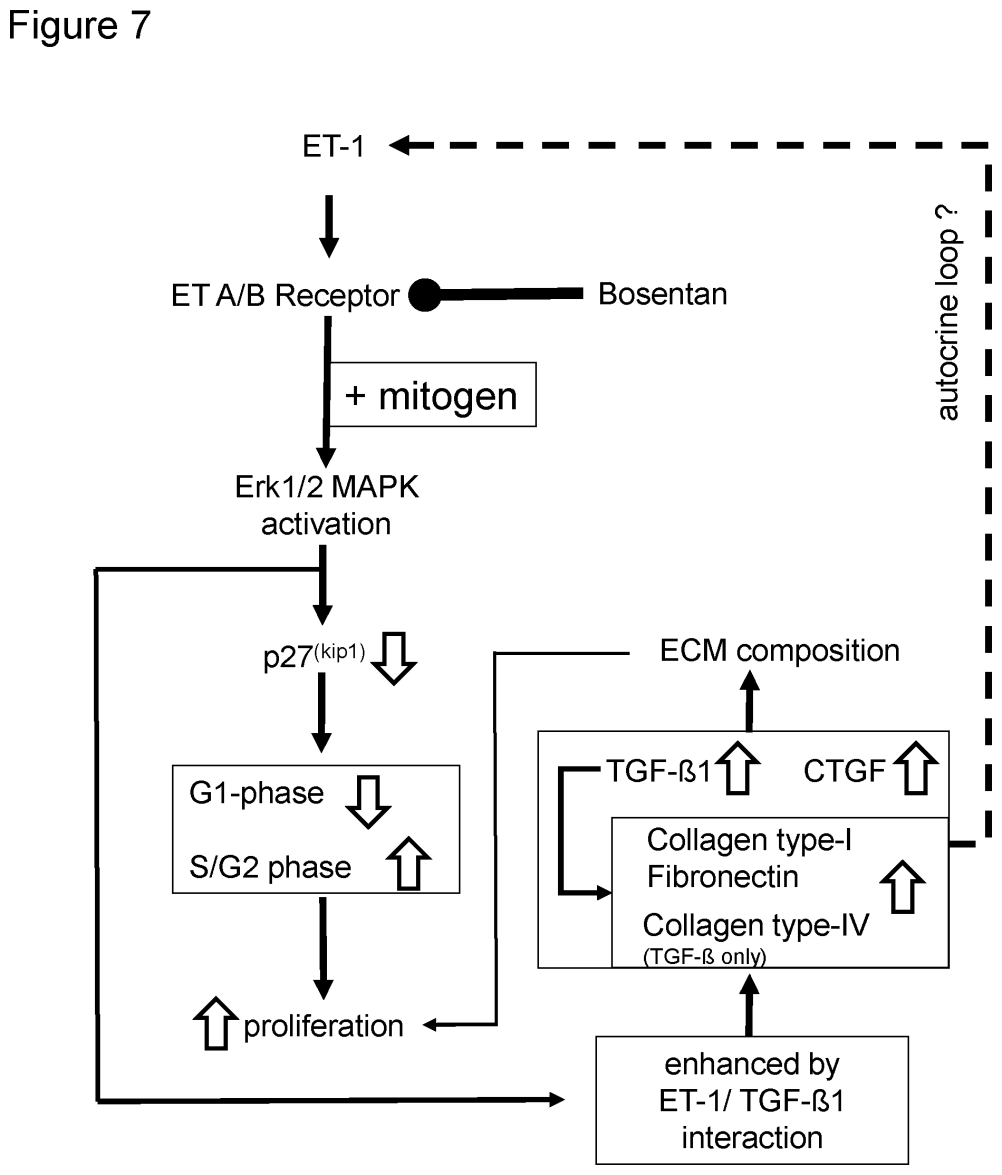
Hypothesis of the role of ET-1 in vessel wall remodeling in PAH. ET-1 alone is a weak inducer of vessel wall remodeling in PAH, but it supports the effect of other mitogens. The supportive effects of ET-1 on vessel wall remodeling involve Erk1/2 MAPK and for ECM deposition TGF-β1 as well as CTGF, but in an ECM component specific mechanisms. We also suggest two major feedback mechanisms: which are activated by ET-1: (a) modified the ECM composition (increased collagen type-I and fibronectin) prepare a vessel wall condition of progressive inflammation; and (b) increased TGF-β1 and collagen type-I stimulate the release of more ET-1 (dotted line: not yet confirmed). The observation that Bosentan or ET-1 receptor blockade abolished all pathological effects of ET-1 suggests that long-term treatment may result in improved symptoms.

PASMC play a key role in the pathology of vascular remodeling in PAH. Antagonism of ET-1 receptors is established as one of the most effective PAH therapies [13]. Our study shows that ET-1 has no mitogenic effect on human PASMC. However, in the presence of inflammatory stimuli such as serum, ET-1 increases cell proliferation. Bosentan reduces this ET-1 effect through up-regulation of p27^(Kip1)^. ET-1 increased the *de novo* synthesis and deposition of collagen type-I involving TGF-β1 and for fibronectin also CTGF. The increase of both ECM components by ET-1 was inhibited by Bosentan. The hypothesis that arterial wall remodeling in PAH results from the supportive effect of ET-1 signaling on that of other mitogens [11] and on TGF-β1 induced collagen deposition via ERK1/2 MAPK is summarized in Figure 7.

Our data show that ET-1 up-regulates PASMC proliferation only in the presence of other mitogens such as serum, which could be used as a model for inflammatory conditions [22,23]. The intracellular signaling pathway that mediates synergistic pro-proliferative effects of ET-1 involves ERK1/2 MAPK, which has been reported earlier to contribute to the remodeling of the neo-intima in different diseases including PAH [11,16,19]. Interestingly, the activation of ERK1/2 MAPK by ET-1 does not occur within minutes, but only becomes significant after 1 hour and lasts over the next 5 hours. The proliferative effect as well as the activation of ERK1/2 MAPK is dependent on ET-1 receptor activity since pre-incubation with Bosentan blocked both effects, which is in line with other studies [11]. Moreover, the activation of ERK1/2 MAPK in vascular smooth muscle cells in particular, was linked to p27^(Kip)^ activation and specific cellular compartmental accumulation [27,28]. In lung tissue, the over-expression of p27^(Kip)^ was associated with attenuated systemic arterial smooth muscle cell proliferation [29]. Our observation that Bosentan inhibits ERK1/2 MAPK activity and increases p27^(Kip)^ expression may explain the observed reduction of vessel wall thickening, which has been documented in clinical studies [30].

As mentioned above, vessel wall remodeling in PAH consists of two major components, proliferation of tissue forming cells and increase deposition of ECM [17,18,31]. In our experiments, ET-1 increased the *de novo* synthesis and secretion of both factors, with TGF-β1 preceding that of CTGF, similar to the sequence reported in dermal fibroblasts [32]. In rat vascular smooth muscle cells, though, ET-1 up-regulates CTGF independent of TGF-β1 [33]. Our data indicate that ET-1 alone is insufficient to increase total ECM deposition significantly, but it clearly supported TGF-β1 dependent ECM deposition. However, when combined, ET-1 further enhanced TGF-β1 induced collagen type-I and fibronectin deposition, a similar effect has been reported in fibroblasts where the effect of the combined stimuli was additive [32]. Importantly, the supportive effect of ET-1 on TGF-β1 that we observed on collagen type-I and fibronectin deposition was prevented by Bosentan, which might further explain the reduced vessel remodeling in PAH animal models [31]. We did not observe any supportive effect of ET-1 on TGF-β1-induced collagen type-III nor type-IV deposition. The discrepancy of the stimulatory effect of ET-1 alone on total ECM versus collagen type-I and fibronectin deposition that we observed may be explained by the different methods used. Total ECM deposition was determined by proline incorporation over the last 24 hours of 48 hours stimulation, while the deposition of specific ECM compounds was determined by in house developed ELISAs after 48 hours.

Similar to fibroblasts, the stimulatory effect of ET-1 alone on collagen type-I deposition involved TGF-β1, but not CTGF [33]. In other smooth muscle cell types and different culture conditions, TGF-β1 and CTGF were necessary to up-regulate collagen type-I deposition [34]. In vascular smooth muscle cells, ET-1 induced collagen type-I synthesis was independent of TGF-β1 but dependent on CTGF [35]. The second ECM compound that was up-regulated by ET-1 through TGF-β1 and CTGF was fibronectin, which has recently been reported to be modified in pulmonary hypertension [36]. On the basis of other reports, it can be speculated that an increase of fibronectin in PAH vessels cells may lead to modified interactions between tissue forming cells and immune reactive cells as well as it altered the response of PASMC to neuronal stimuli [37,38]. The importance of the ECM composition in pulmonary arteries to the pathogenesis of PAH has recently been discussed and it was concluded that this might present a marker for the disease progression. However, too little is known on the precise mechanism [29,39]. Such ECM modifications also might present a feedback mechanism to growth factors and hyperplasia as suggested in Figure 7 [27]. Importantly, the pathological effects of ET-1 were all prevented by Bosentan, which is in line with the beneficial anti-remodeling effect of the drug in a clinical study [30].

In conclusion, our study provides evidence that ET-1 alone is a weak activator of PASMC responses. However, ET-1 significantly supports effects of other PAH relevant stimuli on PASMC responses that could explain PAH vessel wall pathologies. Regarding the therapeutic effect of Bosentan or ET-1 receptors inhibition in general, our data suggests a highly specific beneficial action of the drug.
